# Medicarpin, a Natural Pterocarpan, Heals Cortical Bone Defect by Activation of Notch and Wnt Canonical Signaling Pathways

**DOI:** 10.1371/journal.pone.0144541

**Published:** 2015-12-11

**Authors:** Manisha Dixit, Ashutosh Raghuvanshi, Chandra Prakash Gupta, Jyoti Kureel, Mohd Nizam Mansoori, Priyanka Shukla, Aijaz A. John, Kavita Singh, Dipak Purohit, Pallavi Awasthi, Divya Singh, Atul Goel

**Affiliations:** 1 Division of Endocrinology and Centre for Research in Anabolic Skeletal Targets in Health and Illness (ASTHI)CSIR-Central Drug Research Institute, B.S. 10/1, Sector-10, Jankipuram Extension, Lucknow, India; 2 Division of Medicinal & Process Chemistry, CSIR-Central Drug Research Institute, B.S. 10/1, Sector-10, Jankipuram Extension, Lucknow, India; 3 Sophisticated Analysis and Instrumentation Facilities, CSIR-Central Drug Research Institute, B.S. 10/1, Sector-10, Jankipuram Extension, Lucknow, India; University of California, Los Angeles, UNITED STATES

## Abstract

We evaluated the bone regeneration and healing effect of Medicarpin (med) in cortical bone defect model that heals by intramembranous ossification. For the study, female Sprague–Dawley rats were ovariectomized and rendered osteopenic. A drill hole injury was generated in mid femoral bones of all the animals. Med treatment was commenced the day after and continued for 15 days. PTH was taken as a reference standard. Fifteen days post-treatment, animals were sacrificed. Bones were collected for histomorphometry studies at the injury site by micro-computed tomography (μCT) and confocal microscopy. RNA and protein was harvested from newly generated bone. For immunohistochemistry, 5μm sections of decalcified femur bone adjoining the drill hole site were cut. By μCT analysis and calcein labeling of newly generated bone it was found that med promotes bone healing and new bone formation at the injury site and was comparable to PTH in many aspects. Med treatment led to increase in the Runx-2 and osteocalcin signals indicating expansion of osteoprogenitors at the injury site as evaluated by qPCR and immunohistochemical localization. It was observed that med promoted bone regeneration by activating canonical Wnt and notch signaling pathway. This was evident by increased transcript and protein levels of Wnt and notch signaling components in the defect region. Finally, we confirmed that med treatment leads to elevated bone healing in pre-osteoblasts by co localization of beta catenin with osteoblast marker alkaline phosphatase. In conclusion, med treatment promotes new bone regeneration and healing at the injury site by activating Wnt/canonical and notch signaling pathways. This study also forms a strong case for evaluation of med in delayed union and non-union fracture cases.

## Introduction

Bone possesses an inbuilt capacity of bone regeneration which is either in response to an injury or as part of skeletal development and bone remodelling[1]. The process of bone regeneration encompasses a series of biological events where a number of cell types, local factor and extracellular matrix work together to restore skeletal function[1]. Bone regeneration process involves continuous remodelling throughout adult life[2]. However, certain situations such as in fracture and trauma and conditions like osteoporosis, bone regeneration is required in large quantity. In the clinical setting, the most common form of bone regeneration is fracture healing [1, 2].

The process of bone healing recapitulates the process of skeletogenesis. Bone healing may be indirect or direct bone healing. Indirect bone healing is the most common form where bone healing occurs by both endochondral and intramembranous ossification. In most clinical cases of bone fracture, both cortex and marrow are disrupted. Bone regeneration in these cases involves endochondral ossification and cortical bone regeneration occurs secondarily. The formation of a cartilaginous callus which later is replaced with bone is the key feature of this process. On the contrary, direct bone healing takes place by intramembranous ossification where pre-osteoblasts directly differentiate into osteoblasts [3]. Cortical bone healing is one such model where cortical gap bridging occurs rapidly by intramembranous ossification [4].

Clinical and experimental studies have demonstrated that bone healing in post menopausal osteoporosis women and estrogen deficient osteoporotic animals is significantly delayed or impaired[5]. Even though a lot of emphasis has been given to develop new pharmacological agents that enhance bone mass, there is paucity of literature reports that aim towards enhancing bone regeneration in osteoporotic conditions. Healing promoting factors such as growth factors are being extensively studied and these include vascular endothelial growth factor (VEGF), TGF-β, PDGF, and BMPs such as BMP-2, BMP-7[6]. In fact recombinant human BMP2 (INFUSE® Bone Graft) has been approved for open tibial fractures by FDA[6]. Studies have also shown that the replenishment of BMP2 in the drill-hole in the bone of vitamin A-deficient mice normalized mRNA expressions of the osteogenic genes and the duration for filling the defect with regenerating bone to the same levels as those in control mice[7]. However, the use of BMP2 is hampered by numerous clinical complications which include postoperative inflammation, cyst-like bone formation and life-threatening cervical swelling. Food and Drug Administration (FDA) has in fact issued a warning that in anterior cervical spine surgery, use of BMP/INFUSE posed the risks of dysphagia, hematoma and swelling ([8, 9]. Other disadvantages include a very high cost, and the requirement to be implanted surgically with a carrier at the site of fracture to have their therapeutic effect[10]. Apart from BMPs, it has been studied that daily systemic injections of PTH enhance fracture healing in several rodent models [11–13]. Study by Jung et al has shown that synthetic matrix made of polyethylene-glycol (PEG) containing a covalently bound peptide of the parathyroid hormone (PTH1-34) enhances bone regeneration. PTH therapy, though, again is very expensive and is associated with risk of osteosarcoma, and safety is a major concern for long term use [14, 15]. Consequently, there is an urgent need to discover safe and economical orally active agents that promote bone healing and regeneration.

Studies in our laboratory showed that Butea monosperma plant extract enriched in isoflavones have potent osteogenic activity [16–18]. Several new pterocarpans including few natural products like medicarpin (med) and cajanin were synthesized and evaluated for the treatment of bone disorder. Med exhibited potent activity by enhancing bone quality in both peak bone mass attainment model and Ovx osteopenic mice model [19–21]. Med treatment enhanced osteoblast differentiation and inhibited osteoclastogenesis [20]. Based on this background data, we envisaged that med may possess bone healing properties. Hence, the bone regenerative capacity of med was evaluated in an Ovx rodent model where cortical defect was generated in femoral mid-diaphysis by drill hole injury. Our study reveals that med treatment repairs cortical bone defect and enhances bone regeneration in Ovx osteopenic rodents by activating notch and Wnt canonical signaling pathways.

## Materials and Methods

### Reagents and chemicals

Cell culture media, supplements and calcein was purchased from Sigma–Aldrich (St. Louis, MO). Antibodies for western blot analysis and immunohistochemistry were obtained from cell signaling technology (Danvers, MA, USA) and Santa Cruz Biotechnology (CA, USA). Human PTH (1–34) was purchased from Calbiochem (Darmstadt, Germany).

### Synthesis of med-

Med was synthesized in three steps with improved yield according to the modified procedure [22]. Briefly, med was prepared by a reaction of resorcinol and 2-(2,4-dimethoxyphenyl) acetic acid in the presence of lewis acid BF_3_-OEt_2_ complex solution at 110°C with subsequent addition of mesyl chloride in DMF to furnish an intermediate 3-(2,4-dimethoxyphenyl)-7-hydroxy-4H-chromen-4-one followed by demethylation od 2’-methoxy group. The product 7-hydroxy-3-(2-hydroxy-4-methoxyphenyl)-4H-chromen-4-one thus obtained was cyclized in the presence of NaBH_4_ in absolute ethanol to afford med in 63% yield as depicted in S1 Fig. The compound was re-crystallized with ethyl acetate in hexane and purity was assessed by the HPLC which was found to be 98.14% (S1 Fig).

### Animals and experimental procedures

The study was carried out in accordance with current legislation of animal experiments (Institutional Animal Ethical Committee at Central Drug Research Institute, Lucknow) obtained from the National Laboratory Animal Centre, CSIR-CDRI. The study was approved by the Institutional Animal Ethical committee at Central Drug Research Institute, Lucknow (CPCSEA Registration number: 34/1999 dated 11.3.99; Approval reference number: IAEC/2012/70N/Renew 02 (175/14) dated 3.12.2014). Adult Sprague–Dawley rats (8–12 weeks old; 180±20 g each) were randomly divided into six equal groups (n = 12 rats/group) as follows: sham operated (ovary intact) + vehicle (gum acacia in distilled water p.o.) Ovx + vehicle, Ovx+10.0 μg/kg/day PTH [23], Ovx+0.5mg/kg/day med, Ovx+1 mg/kg/day med and Ovx+5 mg/kg/day med in gum acacia. While med and gum acacia was given daily by oral gavage, PTH was given daily subcutaneously. For the study, animals were ovariectomized and left for 90 days for osteopenia to develop. Seven rats per group were taken for histomorphometric studies. Rest were used for RNA, protein and immunohistochemical analysis.

### Drill-hole injury in femur

A drill-hole injury was created in sham operated, ovariectomized and med treated groups as described before [24]. Under anaesthetic condition (ketamine and xylazine 80–120 mg/kg:10–16 mg/kg, intramuscular) mid diaphyseal drill hole injury was generated in mid femur by straight incision of front skin. Periosteum was removed to expose femoral bone surface. A drill hole injury of 0.8mm was created in anterior portion of diaphysis, 2.0 cm above knee joint. ketoprofen at 5 mg/kg, s.c. was given as a pain reliever. Treatment started from next day onwards. Med at the doses of 0.5 mg/kg, 1.0 mg/kg, and 5.0 mg/kg was administered orally for 15 days. PTH (10.0 μg/kg/day) was administered as a reference standard. Fluorochrome calcein (20 mg/kg), dissolved in normal saline was given to all animals before twenty four hours of autopsy via intraperitoneal route. Rats were sacrificed under anaesthetic condition. All animals were euthanized by CO_2_ overdose followed by cervical dislocation. Femur bones having drill hole injury were collected and kept in 70% isopropanol for fixing and later subjected to microarchitecture analysis. For assessment of bone micro-architectural parameters at strictly drill hole site, bones were fixed in an acrylic material and 50μ sections were trimmed using isomet bone cutter. Confocal microscope (CarlZeissLSM510Meta) aided with appropriate filters having an inciting wavelength of 485 nm and an analyzing wavelength of 510 nm at original magnification,100 x was used to visualize and measure the calcein binding. 2 fields per specimen were selected. Calcein binding intensity at the drill site as an indication of bone regeneration amount was evaluated by creating z stack images of drill hole site sections at thickness of 10μ each with a resolution of 0.569 pixels per μm with Bits per pixel: 8 (color LUT) using CarlZiessAM4.2 image analysis software [24].

### Micro-computed tomography (μCT)

Micro CT assessment of excised bones was done by using skyscan 1076 CT scanner(Aartselaar, Belgium) using previously published protocol[20, 21]. Bones were cleaned of muscles and soft tissues and undergone X-ray source of 70 kV, 100 mA with a pixel size of 18 μm for scanning. Reconstruction of images was performed using Sky Scan Nrecon software, which aids network-distributed reconstruction carried out on four personal computers running simultaneously. Region of newly generated bone was captured by drawing ellipsoid contour with CT analyzer software. Microarchitectural parameters encompassing bone volume fraction (BV/TV), Thickness of trabecularized spicules (Tb.Th) and specific bone surface (BS/BV) were assessed as described earlier [24].

### Bone strength testing

To determine the biomechanical properties, we subjected the femurs in each group to three-point bending using a bone strength tester model TK 252C (Muromachi Kikai Co. Ltd., Tokyo, Japan). Each hydrated rat femur was horizontally positioned on the fixture (14 mm span). A vertical, rounded point was used to load on drill hole site with the medial side in front and the anterior side down (i.e., bending occurred around the medial-lateral axis). The force displacement curve was recorded during loading of the drill hole site at 3 mm/min. The maximum force needed to break the bone, energy and the stiffness of the samples was recorded.

### Quantitative real-time polymerase chain reaction (qPCR)

For gene expression studies, portion surrounding the drill hole was dissected with a 1-mm margin from the femoral bone. Tissues were harvested and carefully cleaned of muscle and soft connective tissues. The specimen was frozen in liquid nitrogen and crushed into powder. Total RNA was extracted with Trizol. cDNA was synthesized by using 2 μg of total RNA using revert Aidkit (Fermentas, Austin, USA). Syber green chemistry was used for quantitative assessment of mRNAs for β- Catenin (β-cat), GSK-3 β, Dvl, LRP5, FzD, Ihh, Smo, Smad 1, Smad 5, Smad 8, Notch 1, Jagged 1 and β-Actin following a standardized protocol. The design of sense and antisense oligonucleotide primers was based on published cDNA sequences using the Universal probe library (Roche Diagnostics, USA). Primer sequences are given in Table 1. For real-time PCR, cDNA was amplified with Light Cycler 480 (Roche Diagnostics, Indianapolis, IN, USA). β- Actin was used as normalizing gene.

**Table 1:** Primer sequences of various rat genes used for qPCR.

primer	sequence
B CATENIN	F-CATGGGTGGAACACAGCA R-CCCAGTGCACCCTTCAAC
GSK-3 BETA	F-ATCAAGGCACATCCTTGGAC R- ACGGCTACACAGTGCGATT
Dvl	F-CCTACAAATTCTTCTTCAAGTCTATGG R- CTTGGCATTGTCGTCGAA
LRP5	F-CATCCATGCTGTGGAGGA R-TGTCTCGGGCACAAGGAT
FzD	F-TCTCCGTGCTCTACACCGTA R-GGAAGGCCTGCTCATAGAAGT
Smad 1	F-GCAGCCCTTTTCAGATGC R-ATAGGCTGAGAGCCATCCTG
Smad 5	F-GCCTATGGACACAAGCAACA R-AGGCAACAGGCTGAACATCT
Smad 8	F-ACCATTACCGCAGAGTGGAG R-TGAGGGTTGTACTCGCTGTG
Jagged 1	F-CGCCCTCTGAAAAACAGAAC R-ACCCAAGCCACTGTTAAGACA
Notch 1	F-CTGGACCCCATGGACATC R-ACTGTACACACTGCCGGTTG
IHH	F-TGCCTCCCAGAACTGAAAGA R-CTGCAGGGAAGGTCATGTTT
Smo	F-CAGGAGCTCTCCTTCAGCAT R-CATTGAGTTCAAAAGCCAAACC
B Actin	F-CCCGCGAGTACAACCTTCT R-CGTCATCCATGGCGAACT

### Western blotting analysis

For protein analysis, tissues isolated from the drill hole region were used and crushed in liquid nitrogen. Estimation of protein concentration was quantitated by Bradford assay. 40 micrograms of total protein was then resolved by 10% SDS-PAGE gel. After transfer of proteins onto PVDF membranes (Immobilon-P, Millipore, Billerica, MA, USA), blocking was done with 1% BSA. The membranes were probed with β-Catenin, Phospho β-catenin(ser 33/37/Thr 41), GSK-3β, LEF-1,smad 1, psmad1/5/8, Ihh, Smo, Notch 1, Jagged 1, c-myc, Hes-1, p21 and β-Actin antibodies (Cell Signaling Technology, Danvers, MA, USA) followed by incubation with HRP conjugated secondary antibodies (Cell Signaling Technology, Cell Signaling Technology, Danvers, MA, USA). Membranes were developed using an enhanced chemiluminescence kit (Millipore, Billerica, MA, USA) using Image Quant LAS 4000 (GE Healthcare, Little Chalfont, UK).

### Immunohistochemistry

Femurs were removed by disarticulation of the forelimb through the knee joint. After removal of skin and superficial layers of soft tissues, the femurs containing drill hole site were fixed in 4% paraformaldehyde in PBS, decalcified in EDTA, embedded for sectioning and staining with hematoxylin and eosin. Immunohistochemical localization of activated b-catenin, Notch-1, Runx-2 and OCN was carried out using commercially available specific antibodies (cell signalling technology Danvers, MA, USA). Staining was visualized using the Biovision IHC kit per the manufacturer’s suggested protocol. The slides were counterstained with hematoxylin (Sigma–Aldrich (St. Louis, MO).

### Co-immunofluorescence

Femurs with drill hole injury were dissected free of connective tissues, fixed in 4% buffered formalin, decalcified in 1% EDTA and embedded in paraffin. Transverse sections of 5μm were then cut from each sample. Sections were initially deparaffinized using xylene, rehydrated through an ethanol gradient and permeabilized with 0.1% Triton X-100 followed by blocking with 1% BSA. These were then incubated with β-catenin antibody diluted in 0.5% BSA (1:100) at 4°C overnight. After washings in 1× PBS, sections were incubated with Alexaflour 488 goat anti-rabbit (1:500). Sections were then washed with PBS and incubated with ALP antibody (1:100) diluted in PBS containing 0.5% BSA, overnight at 4°C under humid conditions. Sections were again washed with PBS and incubated with fluorescent Alexa Fluor-647 donkey anti-goat IgG (H + L) (1:500 dilution in PBS) (Molecular Probes, Carlsbad, CA,USA) at room temperature for 1 hour. Sections were counter stained with DAPI for 15 minutes in dark. After 15 min incubation slides were rinsed with PBS and mounted with antifade mounting media ((life technologies, Carlsbad, CA, USA). Sections were visualized under Cell Imaging Station (life technologies, Carlsbad, CA, USA).

### Statistical analysis

Data are expressed as mean ±SEM. The data obtained in experiments with multiple treatments were subjected to one way analysis of variance followed by the Newman-Keuls test of significance using Prism version 3.0 software.

## Results

### Med promotes bone regeneration at the injury site

**Fig 1:**
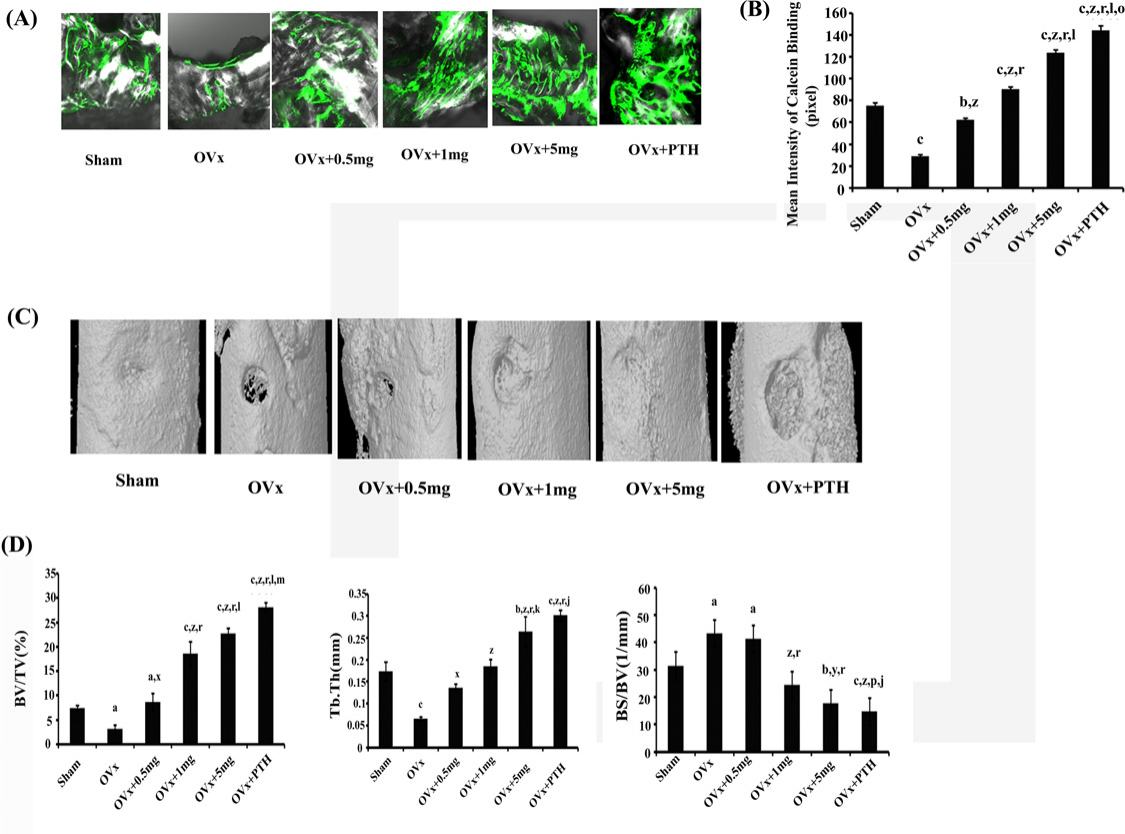
Med promotes bone regeneration at the drill hole site in Ovx osteopenic rats. **(A)** Representative confocal images (100X) of calcein labeling shown in the drill holes of various groups fifteen days after injury. **(B)** Quantification of the mean intensity of calcein label. **(C)** Representative 3-D μCT images from the center of the bony hole in various groups. **(D)** BV/TV, Tb.Th were decreased in the Ovx in comparison to the sham and treatment with med and PTH significantly increased these parameters over the Ovx group. Compared with the sham, BS/BV was increased in Ovx rat and treatment with med and PTH reduced it. N = 7 rats/group; data expressed as mean ±SEM with 95% confidence interval. Statistical analysis was performed by one way-ANOVA nonparametric method followed by the Newman–Keuls test of significance using Prism version 3.0 software. ^c^ *P*< 0.001 compjared with Sham + vehicle group; ^b^ *P*< 0.01compared with Sham + vehicle group; ^a^ *P*< 0.05compared with Sham + vehicle group; ^z^ *P*< 0.001 compared with Ovx + vehicle group; ^y^ *P*< 0.01 compared with Ovx + vehicle group; ^x^ *P*< 0.05 compared with Ovx + vehicle group; ^r^ *P*< 0.001 compared with Ovx + 0.5mg/kg med group; ^p^ *P*< 0.05 compared with Ovx + 0.5mg/kg med group; ^l^ *P*< 0.001 compared with Ovx + 1.0mg/kg med group; ^k^ *P*< 0.01 compared with Ovx + 1.0mg/kg med group; ^j^ *P*< 0.05 compared with Ovx + 1.0mg/kg med group; ^o^ *P*< 0.001 compared with Ovx + 5.0mg/kg med group; ^m^ *P*< 0.05 compared with Ovx + 5.0mg/kg med group.

Effect of med on regeneration of bone was quantified by calcein label (mineral deposition) at the site of drill hole in femur mid-diaphysis. It was observed that compared with Ovx controls (rats receiving vehicle), med at all the three doses of 0.5, 1.0 and 5.0 mg /kg increased new bone formation (measured from the intensity of calcein labeling in the drill hole) in a dose dependent manner (p < 0.001) with 5.0mg/kg dose exhibiting the best effect. Though, the effect was better than the sham group but less robust than PTH, which was used as a reference standard (Fig 1A and 1B).

μCT scans were also performed at the drill hole site. Representative 3-D images are shown in Fig 1C and representative 2-D images are shown in S2 Fig. In comparison to Ovx control, rats treated with med at 0.5mg/kg/d (p < 0.05), 1.0 mg/kg/d (p < 0.001) and 5.0mg/kg/d (p < 0.001) exhibited increased bone volume fraction (BV/TV) in a dose dependent manner (Fig 1D). Increase at 1.0mg/kg and 5.0mg/kg doses was significantly better than 0.5mg/kg dose and sham group but less than the reference standard PTH. Thickness of trabecularized spicules (Tb.Th) within the defect area was also significantly increased at all the three doses in med treated Ovx rats compared to Ovx control (p < 0.05 at 0.5mg/kg dose; p < 0.001 at 1.0 and 5.0 mg/kg doses). Effect at 5 mg/kg dose of med was comparable to PTH and better than sham (Fig 1D). Specific bone surface (BS/BV) was higher in Ovx animals however a significant reduction was observed in Ovx animals treated with med at 1.0 and 5.0 mg/kg/d (Fig 1D).

### Med increases bone mineral density and bone strength at the site of newly generated bone

Assessment of bone mineral density at site of new bone generation surrounding the drill hole site revealed significant increase in BMD in Ovx rats treated with med at dose of 5.0mg/kg and this increase was comparable with PTH (Table 2). Additionally, biomechanical strength was tested at the drill hole site, and parameters like power, energy and stiffness were measured. It was observed that med treated Ovx rats exhibited increased power and energy at the drill site in a dose dependent manner. The increase observed at 5.0mg/kg dose was comparable to PTH. Stiffness was also increased in med treated rats in a dose dependent manner albeit less than PTH treated group (Table 2).

**Table 2:** 

	Sham+vehicle	OVx+ vehicle	OVx +medicarpin (0.5mg/kg)	OVx+medicarpin (1mg/kg)	Ovx+medicarpin (5mg/kg)	Ovx+PTH (10ug/kg)
**POWER(N)**	163.33±2.60	118.66±1.45	138±2.08	146.66±2.60	174±2.88	181.66±1.45
**ENERGY(mJ)**	41.9±1.33	25.99±0.78	29.96±0.87	34.96± 2.60	46.93± 2.88	52.3±1.45
**STIFFNESS** (**N/mm)**	458.33±12.73	272.33±7.79	312.66±5.92	435.33±4.09	499.66±7.79	517.66±5.81
**BMD(g/cm** ^**3**^ **)**	1.016±0.06	0.88± 0.05	0.99±0.03	1.01±0.01	1.16±0.05	1.23±0.05

Values represent mean ± S.E.M. of at least 8 observations in each treatment group

cP<0.001,bP<0.01,aP<0.05 compared to Sham

zP<0.001,yP<0.01,xP<0.05 compared to OVx

rP<0.001,qP<0.01,pP<0.05 compared to OVx+0.5 mg

lP<0.001,kP<0.01,jP<0.05 compared to OVx+1 mg

oP<0.001,nP<0.01,mP<0.05 compared to OVx+5 mg

All other comparisons are statistically non-significant.

### Med promotes expression of osteogenic markers at site of new bone regeneration

**Fig 2:**
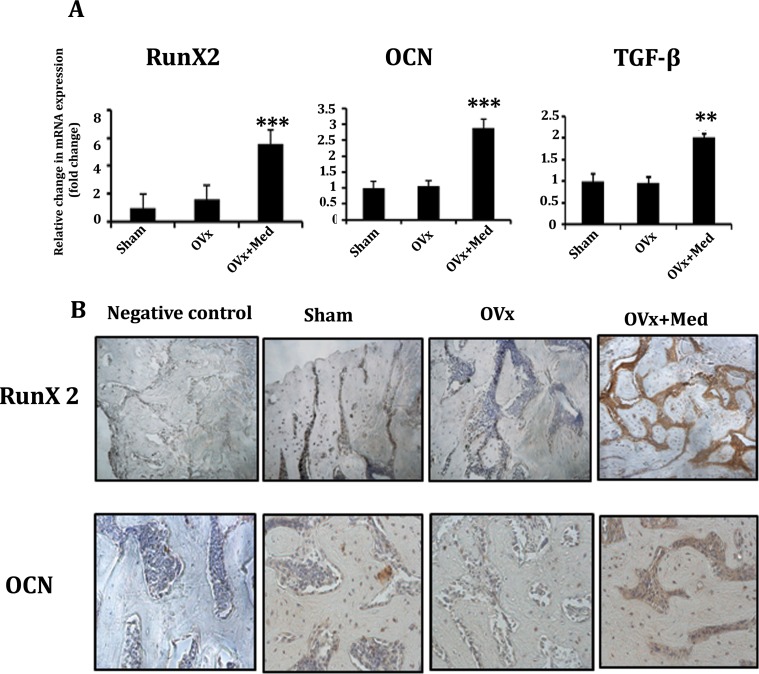
Med treatment increases the expression of osteogenic markers at the site of new bone regeneration. **(A)** mRNA transcript levels of Runx-2, OCN and TGF-β. Data represent three independent experiments and expressed as mean ± SEM with 95% confidence interval. Statistical analysis was performed by ANOVA method followed by the Newman–Keuls test of significance using Prism version 3.0 software. ***P< 0.001 compared with Ovx + vehicle group; **P< 0.01compared with Ovx + vehicle group. **(B)** Med treatment to Ovx rats promotes immunohistochemical localization of Runx-2 and OCN in the area surrounding the drill hole injury area compared to Sham and Ovx control groups.

In order to confirm if rapid bone healing in med treated Ovx rats is attributed to increased osteogenic signals at the injury site, mRNA levels of Runx-2, Osteocalcin (OCN) and TGF-β were determined in regenerating bone. It was observed that med treatment to Ovx rats led to significant increase in transcript levels of Runx-2, OCN and TGF-β compared to untreated Ovx rats (Fig 2A). Further, immunohistochemical localization of Runx-2 and OCN at the injury site demonstrated predominant localization of Runx-2 and OCN in med treated Ovx rats compared to control (Fig 2B).

### Med promotes the differentiation of osteoprogenitors at injury site by activating notch/canonical wnt signalling pathway

**Fig 3:**
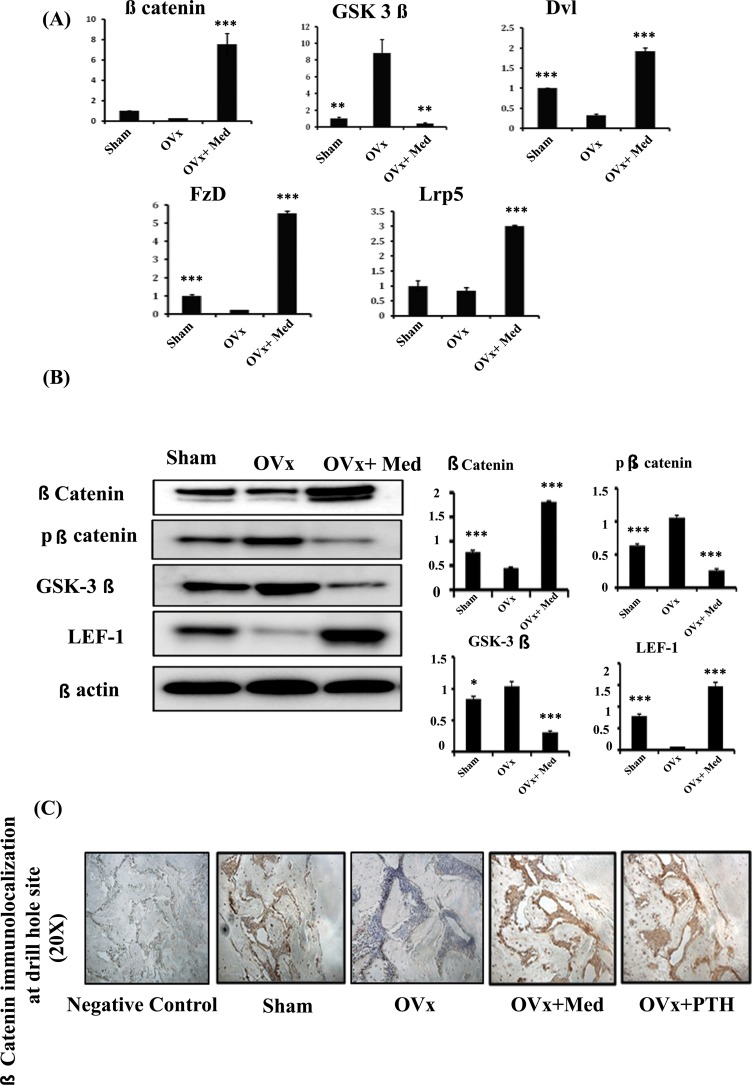
Medicarpin heals cortical bone defects by activating Wnt canonical signaling pathway. **(A)** qPCR analysis of Wnt signaling components like β-catenin, GSK-3β, Dvl, Fzd and Lrp5. **(B)** Western blot analysis and densitometric analyses of phospho and non-phospho β-catenin, GSK-3β and LEF-1. **(C)** Med treatment to Ovx rats promotes immunohistochemical localization of β-catenin in the area surrounding the drill hole injury area compared to Sham and Ovx control groups and was equivalent to PTH treatment group. Data represent three independent experiments and expressed as mean ± SEM with 95% confidence interval. Statistical analysis was performed by ANOVA method followed by the Newman–Keuls test of significance using Prism version 3.0 software. ***P< 0.001 compared with Ovx + vehicle group; **P< 0.01compared with Ovx + vehicle group; *P< 0.05compared with Ovx + vehicle group.

To analyze the mechanism by which med promotes bone healing, tissues at drill hole site were harvested for quantifying mRNA expression levels of different signaling pathways like Ihh (Indian hedgehog), notch, Wnt and BMP signalling. As med at 5.0mg/kg dose gave the best effect, hence tissues were harvested from this group. It was observed that med treatment led to several folds increase in transcripts of Wnt pathway components like β-cat (β-catenin), Dvl (Dishevelled), LRP5 (Lipoprotein related receptor protein-5), Fzd (Frizzled) over Ovx control group and this increase was even more than that observed in sham group (Fig 3A). On the other hand, mRNA levels of Wnt pathways antagonist like GSK3β (Glycogen synthase kinase 3β) were up regulated in Ovx control group and treatment with med inhibited the Ovx induced increase of these inhibitory factors (Fig 3A). As med treatment stimulated the transcript levels of Wnt signaling components, these observations were validated by protein expression and IHC studies. Protein lysates were prepared from tissues surrounding the drill hole injury. It was revealed that med treatment led to increased expression of active β-catenin and transcription factor LEF-1 (Lymphocyte enhancer factor-1) while decreased protein levels of GSK3β, the negative regulator of canonical Wnt signaling (Fig 3B). This was further confirmed by immunohistochemical localization of β-catenin at the bone regeneration site which showed increased accumulation of beta catenin in med treated Ovx rats, compared to Ovx control and sham group and was comparable to PTH treated groups (Fig 3C).

**Fig 4:**
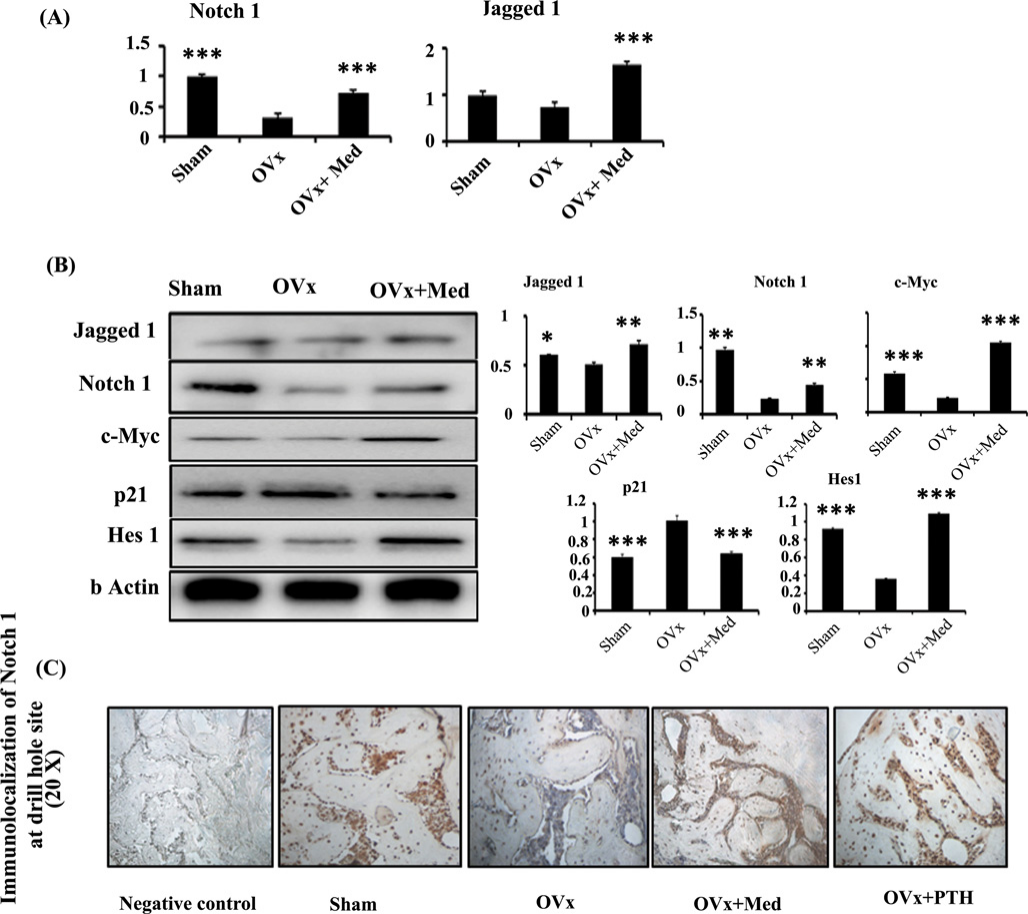
Med also activates notch signaling to induce new bone regeneration at the defect site. **(A)** qPCR analysis of notch signaling components like Notch-1 and Jagged-1. **(B)** Western blot analysis and densitometric analyses of Notch-1, Jagged-1 and notch target genes c-myc, p21 and Hes-1. **(C)** Med treatment to Ovx rats promotes immunohistochemical localization of Notch-1 in the area surrounding the drill hole injury area compared to Sham and Ovx control groups and was equivalent to PTH treatment group. Data represent three independent experiments and expressed as mean ± SEM with 95% confidence interval. Statistical analysis was performed by ANOVA method followed by the Newman–Keuls test of significance using Prism version 3.0 software. ***P< 0.001 compared with Ovx + vehicle group; **P< 0.01compared with Ovx + vehicle group; *P< 0.05compared with Ovx + vehicle group.

Med treatment also stimulated transcript levels of Jagged-1 and Notch-1, key notch signalling mediators (Fig 4A). These observations were further corroborated by protein expression studies. Med treatment led to induction of Notch-1 and Jagged-1 protein expression over Ovx control group, though the expression level of Notch-1 was less than the sham group (Fig 4B). However, expression levels of notch target genes like c-myc, p21 and Hes-1 were significantly more than Ovx group and comparable to sham group (Fig 4B). These results were confirmed by increased localization of Notch-1 at injury site in med treated Ovx rats and were comparable to sham and PTH treated groups (Fig 4C). IHH and Smad signaling pathway appears not to play a substantial role in bone regenerative capacity of med as evaluated by transcript and translational studies (S3 Fig). As previous studies in our laboratory show that med stimulates osteoblast differentiation by augmenting ER/p38MAPK pathway, hence translational levels of ER-β and p38MAPK were determined in all the groups. Protein expression levels of both ER-β and p38MAPK were not different amongst the three groups (S4 Fig). Altogether these observations suggest that notch/canonical wnt signalling may be playing an important role in stimulatory effect of med on bone regeneration.

**Fig 5:**
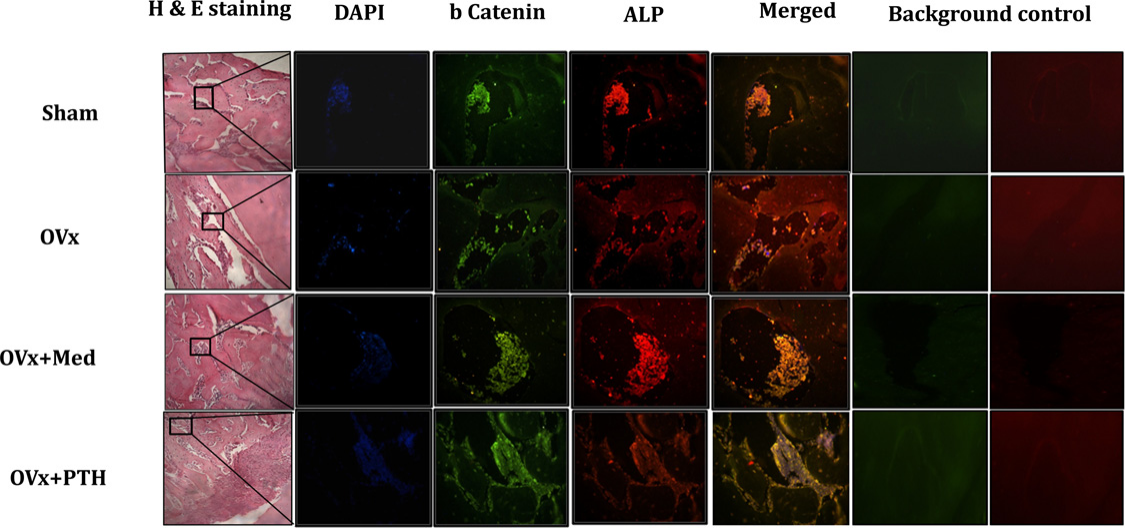
Hematoxylin and eosin staining of newly regenerated bone at the injury site and immunoflourescence analysis of the co localization of β-catenin and ALP in femoral bone sections at the injury site. Immunofluorescence staining of β-catenin (green) and ALP (red) was performed. Co localization of two molecules is demonstrated in merged yellow image. Cells were counterstained with DAPI (blue). Auto fluorescence images were taken in red and green channel for each panel for normalizing the background.

Finally, in order to confirm that med treatment leads to elevated levels of wnt and notch signaling components in pre-osteoblasts and not other adjacent cell types, beta-catenin was co localized with alkaline phosphatase, which is an osteoblast marker, by immunofluorescence. Immunofluorescence analysis revealed an intense staining for beta-catenin at injury site. Single labeling for ALP displayed a similar pattern. Double labeling for beta catenin and ALP demonstrated visual co-localization between the antibodies (Fig 5). This confirmed that med induced Wnt and notch signaling is specific to osteoprogenitors. Additionally, histological analysis showed a larger area of regenerating bone in Ovx animals treated with med, same as that of Sham and PTH treated groups (Fig 5).

## Discussion

In this study we have used the the drill hole injury model to investigate bone regeneration between Ovx rats and med treated Ovx rats. In this model, an injury is created in cortical bone at femur mid-diaphysis. This model is different from normal fracture healing process which occurs by endochondral ossification [3, 25]. Normal fracture healing consists of three phases which are inflammatory and the granulation phase, reparative phase consisting of cartilage callus formation and eventually the remodeling phase to restore normal cortical structure [3, 25]. However, bone healing in drill hole defect model mainly occurs by intramembranous ossification, providing more stable bone regeneration by eliminating chondrocyte differentiation [26, 27]. Moreover, a drill-hole defect in the femur diaphysis does not require a metal fixation device and is thus a simple and highly reproducible model to work with [27].

A drill hole injury of 0.8mm diameter was generated in femoral mid-diaphysis of osteopenic rats in this model and the characteristics of bone healing were intramembranous dominant. Fluorochrome labeling study shows that compared with the sham-operated group, regeneration of new bone in the drill hole was much reduced in Ovx rats, which could be due to the reduced osteoblast function under estrogen deficiency. It was observed that med treatment of Ovx rats significantly augmented the process of filling up of newly generated bone in the drill hole in a dose dependent manner with 5.0mg/kg dose being the most effective although less than the positive reference standard, PTH. This finding was complemented by microCT analysis of the defect area where an increased bone volume fraction was observed in med treated rats. The increase in regenerated bone was largely due to the thickness of trabecularized spicules as Tb.Th within the defect was significantly greater in treated rats. These data indicate that med accelerates the bone-healing process.

Bone mineral density and bone strength were also measured at the site of newly generated bone surrounding the drill hole injury site. It was found that med treatment to Ovx rats led to increased bone mineral density at a dose of 5.0mg/kg dose and the effect was similar to PTH treated Ovx rats. Corroborating this data, increased bone strength parameters (power, energy and stiffness) were observed after med treatment to Ovx rats with best effect observed at 5.0mg/kg body weight dose and this was comparable with PTH.

In order to confirm that increased bone regeneration at injury site in med treated Ovx rats is due to enhanced osteoblastogenesis, transcript levels of bone anabolic markers like Runx-2, OCN and TGF-β were determined which in turn were found to be significantly enhanced compared to control Ovx and sham rats. These observations were corroborated by predominant localization of Runx-2 and OCN at the site of new bone regeneration in med treated Ovx rats. These results also indicated that med induced healing of the femoral cortical defect is mainly through intramembranous ossification.

PTH effects on bone repair are found to be mediated in part through the activation of Wnt-signaling pathways [11]. Hence, we wanted to study the mechanism by which med stimulates bone healing. A number of pathways like BMP and Wnt signalling have been demonstrated to be required for fracture healing and bone regeneration [28]. Our studies indicated that BMP-2 and ER/P38MAPK signaling pathways do not play a part in med induced bone regeneration which was in contrast to our earlier studies with medicarpin effect on osteoblasts. A recent study shows that while BMP-2 plays an important role in endochondral ossification, it is not critical for the process of intramembranous ossification [29]. The canonical Wnt pathway is also well recognized for its role in skeletal development, maintenance of bone mass and bone regeneration [30]. Sclerostin deficient mice have been shown to rapidly heal bone defects by activating β-catenin and increasing intramembranous ossification [30]. It was observed that while med treatment significantly up regulated various Wnt pathway components like β-catenin, Dvl, LRP5 and Fzd [31] over Ovx control group, mRNA levels of Wnt pathway antagonist like GSK3β [31] was significantly decreased compared to the Ovx control group. This observation was interesting as to how Wnt inhibits GSK3 activity towards b-catenin is still not clear. Recent studies have proposed a new cell-biological model according to which Wnt proteins induce the uptake of GSK3 into multivesicular bodies (MVBs) [32]. This event sequesters the enzyme away from newly synthesised b-catenin substrate in the cytoplasm, thus blocking its phosphorylation. Role of wnt signaling was further validated by protein expression studies where med treated Ovx rats exhibited increase in active β-catenin and transcription factor LEF-1 at injury site while GSK3β which is a negative regulator of canonical Wnt signaling was decreased at the site of new bone regeneration. Increased accumulation of β-catenin in the region surrounding the drill hole site in med treated Ovx animals, as assessed by immunohistochemical localization additionally strengthened these results.

Besides Wnt and BMP signaling pathways, Ihh and Notch are also reported to play a role in both endochondral and intramembranous ossification [33, 34]. Interestingly, med treatment though had no effect on Ihh signaling but it led to increased mRNA expression of Notch-1 and Jagged-1. Notch signalling is shown to be up regulated during intramembranous repair [34]. Activation of notch signalling occurs when one of the notch ligands, Jagged 1,2 and Delta-like 1,4 interacts with one of notch receptor notch 1–4 which subsequently initiates the notch signalling pathway [34]. Med treatment to Ovx rats led to increased protein expression of Notch-1, Jagged-1 and notch target genes like c-myc, Hes-1 and p21 in region surrounding the drill hole injury. This potentiated the role of notch signalling in med mediated repair of cortical bone defects. These results were further corroborated by enhanced localization of Notch-1 at the site of drill hole injury in med treated Ovx rats. Together, these results clearly suggest that med accelerates bone healing by activation of notch and Wnt canonical signaling pathway. There are reports that Wnt signaling by repressing GSK-3β may lead not only to the accumulation of β-catenin and Wnt target genes but also to the accumulation of the intracellular fragment of notch and activation of notch targets such as Hes1[35, 36]. However, there is always the possibility that both Wnt and notch may represent parallel pathways in the bone healing effect presented by med. Finally, to confirm that Wnt and Notch signaling were driving differentiation of osteoprogenitors at the site of injury, co localization of beta catenin was carried out with ALP, which is a marker for osteoblast differentiation. It was observed that both beta catenin and ALP co localized together indicating that med led to increased bone regeneration by inducing Wnt and notch signaling in pre-osteoblasts.

In summary, our data shows that med promotes bone healing by accelerating the intramembranous repair of cortical bone defects. The bone healing effect of med is attributed to activation of notch and Wnt canonical signaling pathways which are known to increase intramembranous ossification. This study also forms a strong case for evaluation of med in delayed union and non-union fracture cases.

## Supporting Information

S1 Fig Synthesis scheme and HPLC chromatogram of medicarpin. (TIF)

S2 Fig Representative 2-D μCT images from the centre of the bony hole in various groups. (TIF)

S3 Fig Ihh and Smad signaling pathways do not play a role in bone healing effect of medicarpin. (A) qPCR analysis, (B) western blot analysis and densitometry data. Data represent three independent experiments and expressed as mean ± SEM with 95% confidence interval. Statistical analysis was performed by ANOVA method followed by the Newman–Keuls test of significance using Prism version 3.0 software. ***P< 0.001 compared with Ovx + vehicle group; **P< 0.01compared with Ovx + vehicle group; *P< 0.05compared with Ovx + vehicle group.
(TIF)

S4 Fig Protein expression levels of ER-β and p38MAPK was determined by western blot analysis. These remain unchanged in the tissue harvested from med treated Ovx group. Data represent three independent experiments and expressed as mean ± SEM with 95% confidence interval. Statistical analysis was performed by ANOVA method followed by the Newman–Keuls test of significance using Prism version 3.0 software.
(TIF)
