# Supplementary figures and images for: Medicarpin, a Natural Pterocarpan, Heals Cortical Bone Defect by Activation of Notch and Wnt Canonical Signaling Pathways
Source: PLoS One. 2015 Dec 11;10(12):e0144541. doi: 10.1371/journal.pone.0144541 (PMC4676632; doi:10.1371/journal.pone.0144541)

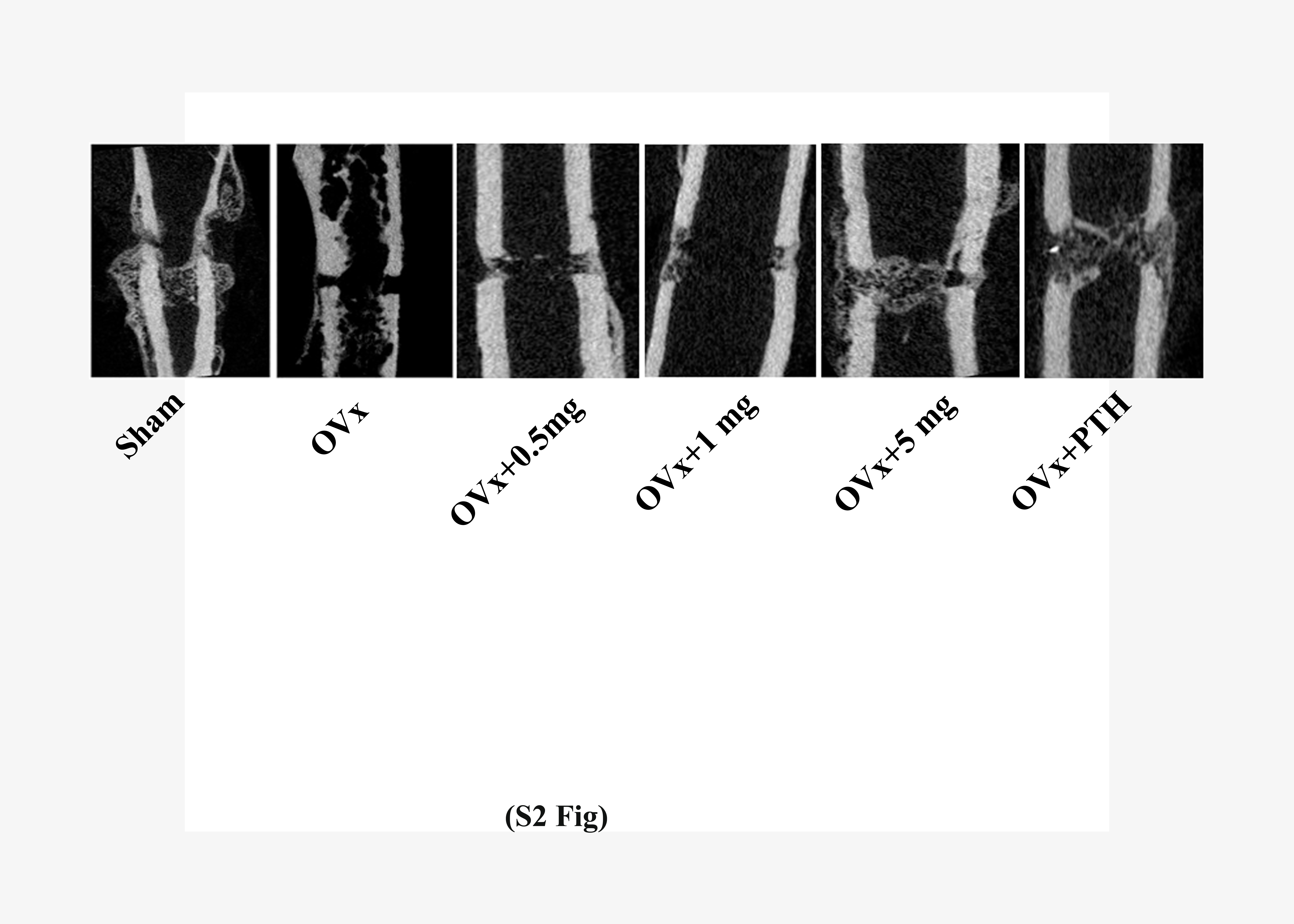

Supplement: S2 Fig — (TIF) [file pone.0144541.s002.tif]
